# Molecular Characterization of a Chrysovirus Isolated From the Citrus Pathogen *Penicillium crustosum* and Related Fungicide Resistance Analysis

**DOI:** 10.3389/fcimb.2019.00156

**Published:** 2019-05-15

**Authors:** Shengqiang Wang, Zhu Yang, Tingfu Zhang, Na Li, Qianwen Cao, Guoqi Li, Yongze Yuan, Deli Liu

**Affiliations:** ^1^Hubei Key Laboratory of Genetic Regulation and Integrative Biology, School of Life Sciences, Central China Normal University, Wuhan, China; ^2^College of Life Science and Technology, Honghe University, Mengzi, China

**Keywords:** molecular characterization, chrysovirus, citrus, pathogen, *Penicillium crustosum*

## Abstract

*Penicillium* sp. are damaging to a range of foods and fruits including citrus. To date, double-stranded (ds)RNA viruses have been reported in most *Penicillium* species but not in citrus pathogen *P. crustosum*. Here we report a novel dsRNA virus, designated as *Penicillium crustosum* chrysovirus 1 (PcCV1) and isolated from *P. crustosum* strain HS-CQ15. PcCV1 genome comprises four dsRNA segments, referred to as dsRNA1, dsRNA2, dsRNA3, and dsRNA4, which are 3600, 3177, 3078, and 2808 bp in length, respectively. Sequence analysis revealed the presence of four open reading frames (ORFs) in the PcCV1 genome. ORF1 in dsRNA1 encodes a putative RNA-dependent RNA polymerase (RdRp) and ORF2 in dsRNA2 encodes a putative coat protein (CP). The two remaining ORFs, ORF3 in dsRNA3 and ORF4 in dsRNA4, encode proteins of unknown function. Phylogenetic analysis based on RdRp sequences showed that PcCV1 clusters with other members of the genus *Chrysovirus*, family *Chrysoviridae*. Transmission electron microscope (TEM) analysis revealed that the PcCV1 visions are approximately 40 nm in diameter. Regarding biological effects of PcCV1, HS-CQ15 harboring the chrysovirus exhibited no obvious difference in colony morphology under fungicide-free conditions but decreased resistance to demethylation inhibitor (DMI)-fungicide prochloraz, as compared to PcCV1-cured strain. Here we provide the first evidence of a virus present in citrus pathogenic fungus *P. crustosum* and the chrysovirus-induced change in fungicide-resistance of its host fungus.

## Introduction

Mycoviruses have been widely distributed in fungal hosts including various *Penicillium* species. Mycoviruses with double stranded RNA (dsRNA) genomes can be categorized into seven families, as reviewed by Ghabrial et al. (2015), i.e., *Totiviridae, Chrysoviridae, Partitiviridae, Reoviridae, Megabirnaviridae, Quadriviridae*, and *Endornaviridae* families. Among these dsRNA mycoviruses, the members of family *Chrysoviridae* have been early identified in *P. chrysogenum* (Lemke and Ness, 1970; Lemke et al., 1973; Yamashita et al., 1973; Edmondson et al., 1984; Jiang and Ghabrial, 2004), later in *C. nitschkei* (Liu et al., 2007) and rice pathogenic fungus *Magnaporthe oryzae* (Urayama et al., 2010), and recently in filamentous phytopathogenic fungus *Colletotrichum gloeosporioides* (Zhong et al., 2016), *Brassica campestris* var. purpurea (Zhang et al., 2017), entomopathogenic fungus *Isaria javanica* (Herrero, 2017) and *Alternaria* species (Okada et al., 2018). Most of the chrysoviruses reported to date constitute family *Chrysoviridae* that can be classified into two clades, and the members in clade II constitute genus *Chrysovirus* (Liu et al., 2012). However, the evidence on inhabitant of any chrysovirus (even any mycovirus) in host fungus *P. crustosum* is still lacking.

Members of the family *Chrysoviridae* share some common traits regarding their dsRNA genome structures as intensively reviewed before (Ghabrial, 2010; Ghabrial et al., 2015). Usually, a typical chrysovirus genome is comprised of 4 segmented dsRNAs in 2.4~3.6 kbp full-length, separately encapsidated to form virion particles in ~40 nm size (Ghabrial et al., 2018). For tetra-segmented genome of chrysovirus, dsRNA1 as the largest segment encodes RNA-dependent RNA polymerase (RdRP), often referred to as P1 in literatures (Jiang and Ghabrial, 2004; Ghabrial et al., 2018), exhibiting 8 conserved motifs found in most dsRNA viruses inhabiting lower eukaryotes (Bruenn, 1993), dsRNA2 encodes the major capsid protein (CP), often referred to as P2 (Jiang and Ghabrial, 2004; Ghabrial et al., 2018), and the rest two dsRNAs (dsRNA3 and dsRNA4) encodes unknown-function proteins, i.e., P3 and P4, respectively (Ghabrial et al., 2018). Sequence-based predictions indicate P3 contains a phytoreovirus S7 domain and has sequence similarity with the RdRP at its N-terminus, and P4 is a putative protease (Liu et al., 2012). Significantly high sequence identity has been observed at both 3′- and 5′-UTRs of chrysovirus genomic dsRNAs, including 5′- and 3′-terminal sequences strictly conserved (Ghabrial, 2010), 40–75 nt region conserved at 5′-UTRs (Ghabrial, 2010; Herrero, 2017), and CCA-repeats in the 30–50 nt stretch at 5′-UTRs (Jiang and Ghabrial, 2004; Urayama et al., 2010; Zhang et al., 2017; Okada et al., 2018). To date, some unusual genome structures composed of 5 or 3 dsRNA segments were documented for chrysoviruses infecting *Fusarium graminearum* (Darissa et al., 2011; Yu et al., 2011), *M. oryzae* (Urayama et al., 2012, 2014), radish *Raphanus sativus* (Li et al., 2013), and *Brassica campestris* (Zhang et al., 2017). These viruses are closely related to recognized chrysoviruses, nevertheless, they are not officially accepted species yet. Now that such great diversity of chrysoviruses that may beyond expected, it would be an interesting issue to identify mycovirus(es) in citrus pathogen *P. crustosum*.

We have reported a number of novel mycoviruses in *P. digitatum* species, including *Penicillium* digitatum virus 1 (PdV1) (a member of the genus Victorivirus in the family Totiviridae) (Niu et al., 2016), *Penicillium* digitatum polymycovirus 1 (PdPmV1, a polymycovirus) and *Penicillium* digitatum narna-like virus 1 (PdNLV1, a narna-like virus) in 2018 (Niu et al., 2018), and *Penicillium* digitatum gammapartitivirus 1 (PdGV1, a partitivirus) in 2018 (Yang et al., 2018). The present report provided the first evidence on a chrysovirus inhabited in *P. crustosum* isolate HS-CQ15, molecularly characterized this mycovirus to the member of *Chrysoviridae* family, referred to as ‘*Penicillium crustosum* chrysovirus 1’ (PcCV1), and effect of PcCV1 infection on the fungal resistance to DMI-fungicide prochloraz was also investigated.

## Method

**Figure 1:**
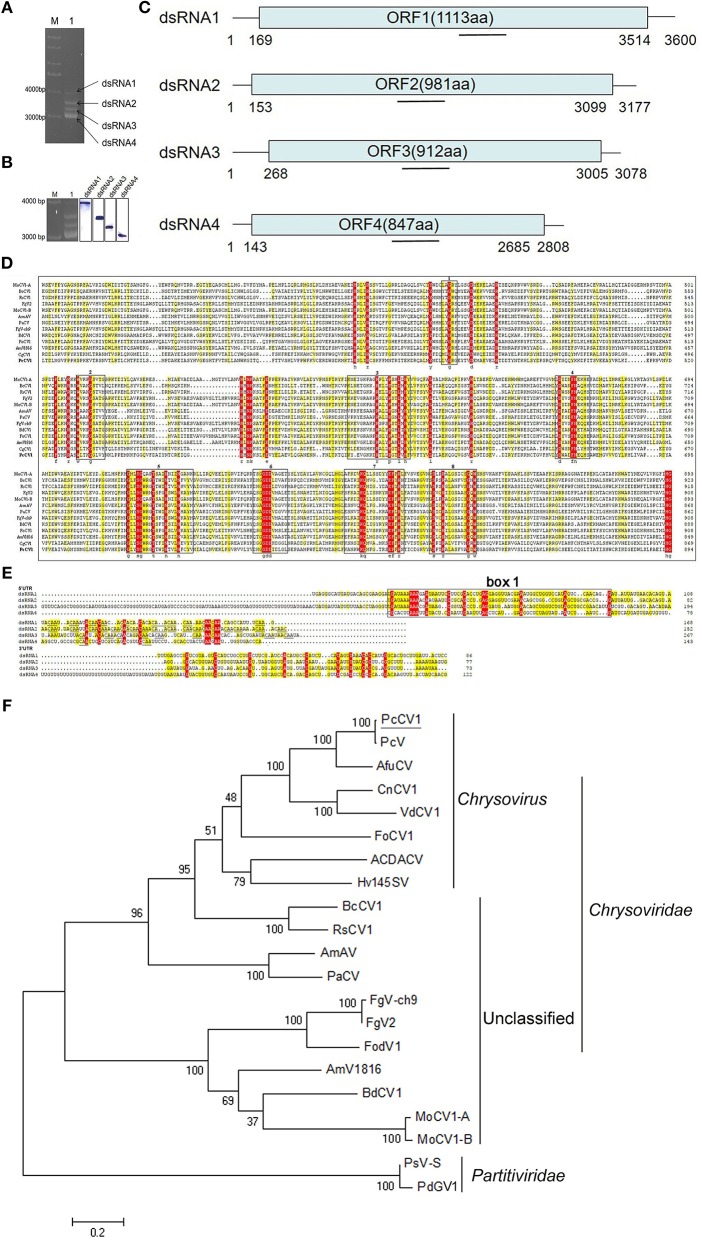
Characterization of PcCV1 genomic organization and phylogenetic analysis. **(A)** Mobility pattern analysis of PcCV1 dsRNAs from *P. crustosum* HS-CQ15 mycelia (lane 1) by agarose gel electrophoresis. Lane M indicates DNA marker DS10000 (TaKaRa, Dalian, China). **(B)** Northern-blot confirmation of PcCV1 genomic dsRNAs. The left panel is the copy of **(A)**, indicating in-gel positions for dsRNA1 to 4 separated as mentioned above. The full-scan of entire original gels for the RNA blots of PcCV1 genomic dsRNAs is shown in Figure S3. **(C)** Schematic representation of PcCV1 genome organization. The boxes represent the ORFs within genomic dsRNAs, and lines depict 5′- and 3′-untranslated regions (UTRs). **(D)** Multiple alignments of RdRps between PcCV1 and other chrysoviriuses. The conserved motifs in the selected RdRp sequences are boxed with numbers 1–8. The names (full and abbreviated) of selected chrysoviriuses as well as GenBank accession numbers of their RdRps are listed in Table S2. **(E)** Nucleotide sequence alignments of 5′- and 3′-UTRs of PcCV1 genomic dsRNAs. The identical nucleotides among dsRNA1 to 4 are especially color-shaded. The “box 1” is highlighted in black box, and the “CAA” repeats are highlighted with underlines. **(F)** Phylogenetic analysis of the RdRp sequences for PdGV1. The names (full and abbreviated) of selected chrysoviriuses as well as GenBank accession numbers of their RdRps are listed in Table S2.

The host of PcCV1, *Penicillium* HS-CQ15, was isolated from *Penicillium*-decayed citrus surface (Chongqing, China) and molecularly characterized as *P. crustosum* species, according to internal transcribed spacer (ITS) analysis described before (Gardes and Bruns, 1993; Pandey et al., 2018). HS-CQ15 conidial suspension stored in glycerol at −70°C was initially cultured on potato dextrose agar (PDA) medium at 28°C and 180 rpm for about 7 days, and the resulting mycelium fragments were transferred into potato dextrose broth (PDB) medium for additional 96 h cultivation at the same conditions. The fungal mycelia collected from the PDB cultures was exploited to extract viral dsRNAs, using phenol-chloroform-ethanol method (Sun and Suzuki, 2008; Sotaro et al., 2009). The obtained dsRNA mixture was purified by DNase I and S1 nuclease digestions at RNase-free conditions, separated by 1% (w/v) agarose gel electrophoresis, and finally recovered from individual EB-stained band using Gel Extraction kit (TaKaRa, Dalian, China). The amount of ~5 μg dsRNA recovered was applied to construct cDNA libraries for Illumina high-throughput sequencing, according to protocols described before (Rwahnih et al., 2011; Niu et al., 2018). Afterwards, reads and contigs assembly, based on reference genome of PcV (Jiang and Ghabrial, 2004), provided sequences with partial length for PcCV1 dsRNAs, i.e., 2437 bp for dsRNA1, 3096 bp for dsRNA2, 2909 for dsRNA3, and 2726 bp for dsRNA4. Thirteen pairs of specific primers (Table S1) were designed to full-fill gaps by RT-PCR, generating ORF-included sequences, and then, 4 pairs of adaptor primers (Table S1) were designed to PCR-amplify 5′- and 3′-UTRs, finally generating full-length PcCV1 genomic dsRNAs. According to the full-length sequences, specific RNA probes were designed as shown in Figure 1C and used for digoxigenin-labeled northern blots, as previously described (Streit et al., 2009; Niu et al., 2018).

Open reading frames (ORFs) in putative viral sequences were identified using the NCBI ORF finder (http://www.ncbi.nlm.nih.gov/gorf/gorf.html) and further confirmed by simulated translation in DNAMAN software package version 6.0 (Lynnon Corporation, Quebec, Canada). The analysis of protein sequence similarity was initially performed using the BLASTp program at NCBI website (http://www.ncbi.nlm.nih.gov/). Multiple sequence alignments were further processed with CLUSTAL_X program (Thompson et al., 1997), and phylogenetic trees were constructed using the neighbor-joining method in MEGA version 6.0 (Tamura et al., 2013), and further confirmed by maximum likelihood approach in the same software.

Viral particles (VPs) were purified and characterized as described previously (Niu et al., 2016, 2018). Approximately 100 g (wet weight) mycelia of HS-CQ15 were harvested from PDB cultures, mixed with 400 mL of 50 mM sodium phosphate buffer (pH 7.4), and ground into homogenates for VPs isolation using sucrose density gradient ultracentrifugation. The obtained VPs were suspended with sodium phosphate buffer (pH7.4) for the use of transmission electron microscopy (TEM) and SDS-PAGE analysis.

To investigate biological effects of PcCV1 on its host fungus, we prepared PcCV1-cured HS-CQ15 progenies by ribavirin protocol as described before (Niu et al., 2016, 2018). In detail, the conidia of HS-CQ15 (PcCV1-infected) were incubated in PDB media containing 100 mmol·L^−1^ ribavirin for 12 h to generate virus-free progenies (PcCV1-cured). Five PcCV1-cured strains were selected and independently subjected to the following experiments each containing three biological replicates. The vegetative growth and ability to resist DMI-fungicide for HS-CQ15 and its PcCV1-cured progenies were assessed on fungicide-free PDA media and 5.0 mg L^−1^ (final concentration) prochloraz-containing PDA media, respectively, according to the methods of Niu et al. (2018), and EC_50_ values against prochloraz for the virus-infected and virus-cured strains were also measured. The average of colony diameters in each independent experiment was used for EC_50_ calculation by SPSS software (version 10.0).

## Results and Discussion

Mycovirus PcCV1 was isolated from *P. crustosum* HS-CQ15 and its genomic segments (viral dsRNAs) were extracted and purified by using phenol-chloroform-ethanol method. Four dsRNA bands as PcCV1 genomic segments were designated to dsRNA1, dsRNA2, dsRNA3, and dsRNA4, according to their increasing electrophoresis mobility (Figure 1A). The tetra-segmented genome of PcCV1 has been confirmed by northern blotting (Figure 1B).

The genome organization of PcCV1 is shown in Figure 1C. The sequence of dsRNA1 is 3,600 bp full-length containing a single open reading frame (referred to as ORF1) that encodes a 1,113-amino-acid (aa) protein with putative molecular weight ~128 kDa. Blastn analysis revealed a high nucleotide sequence similarity (~82%) between dsRNA1 and RdRp-encoding gene of a classical chrysovirus *Penicillium* chrysogenum virus (PcV), the earliest case of *Penicillium*-hosted chrysovirus (Jiang and Ghabrial, 2004). Blastp analysis also showed a high similarity (~98%) in aa sequences between ORF1 and PcV RdRp. dsRNA2 with 3,177 bp size contained an ORF (referred to as ORF2) encoding a 981-aa protein (~108.6 kDa) with ~96% similarity to capsid protein (CP) of PcV. dsRNA3 and dsRNA4 with 3,078 bp and 2,808 bp full-length, respectively, both contained a single ORF, encoding 912-aa and 847-aa protein with estimated molecular weight ~101 and ~95 kDa, respectively. These two relatively smaller proteins exhibited highest similarity to function-unknown proteins encoded by PcV dsRNA3 and dsRNA4, as previously documented (Jiang and Ghabrial, 2004), and also showed considerably high similarity to specific function-unknown proteins reported in other chrysoviruses (Jamal et al., 2010; Cao et al., 2011; Herrero, 2017).

Multiple alignment of the PcCV1 RdRp with other International Committee on Taxonomy of Viruses (ICTV)-registered chrysoviruses belonging to the genus *Chrysovirus* (Jiang and Ghabrial, 2004; Urayama et al., 2010), listed in Table S2, showed eight conserved motifs (Figure 1D), as early verified to be typical structural traits of RdRps for dsRNA viruses in lower eukaryotes (Bruenn, 1993). Between the two closely related chrysoviruses (PcV and PcCV1), the amino acid sequence identities of their dsRNA-encoding proteins (P1, P2, P3, or P4) were summarized in Table S3. Specially, a conserved phytoreovirus S7 domain was observed in the upstream of both P1 (RdRp) and P3 sequences (Figure S1). This agreed to the S7 domain reported in other chrysoviruses (Liu et al., 2012). In addition, we found highly conserved sequences at 5′-UTR for the four PcCV1 genomic dsRNAs. As shown in Figure 1E, about 60 nt located at or close to 5′-termini exhibited conserved among the present 4 dsRNAs, as named “box 1” in other reported chrysoviruses (Ghabrial, 2010). The second conserved region at PcCV1 5′-UTR, locating downstream from the “box 1,” is characterized by a cluster of CAA-repeats (Figure 1E), as reported in almost all chrysoviruses (Jiang and Ghabrial, 2004; Urayama et al., 2010; Zhang et al., 2017; Okada et al., 2018). Such CAA-repeats, frequently identified at 5′-UTRs of chrysoviruses, have been functionally associated with translational enhancer elements for tomaviruses (Gallie and Walbot, 1992), thus presumably contributing to control of genome replication and virion assembly of chrysoviruses that facilitated their persistent infections to fungal hosts (Ghabrial et al., 2018). Figure 1E indicated ~13 nt sequence conserved in 3′-UTRs of PcCV1 genomic dsRNAs. The locations of special conserved sequence at 3′-UTRs have been identified in other chrysovirus genomes (Jiang and Ghabrial, 2004; Jamal et al., 2010; Zhong et al., 2016; Herrero, 2017), nevertheless exhibiting sequence diversities among these different chrysoviruses.

The association of PcCV1 with family *Chrysoviridae* was verified by phylogenetic tree analysis (Figure 1F), based on aa sequences of RdRps between PcCV1 and ICTV-annotated dsRNA chrysoviruses. PcCV1 (with the closest association with PcV) was also closely clustered with other previously reported members of the genus *Chrysovirus*. Under TEM scanning, the purified virions of PcCV1 were isometric in ~40 nm diameter (Figure S2A) that was consistent with other reported chrysoviruses virions in size (Urayama et al., 2010; Ghabrial et al., 2018), also supporting the evolutionary position of PcCV1. Here, the purified virions were confirmed to be extracted from PcCV1 by gel analysis of genomic dsRNA segments (Figure S2B) and SDS-PAGE analysis of CP (Figure S2C).

**Figure 2:**
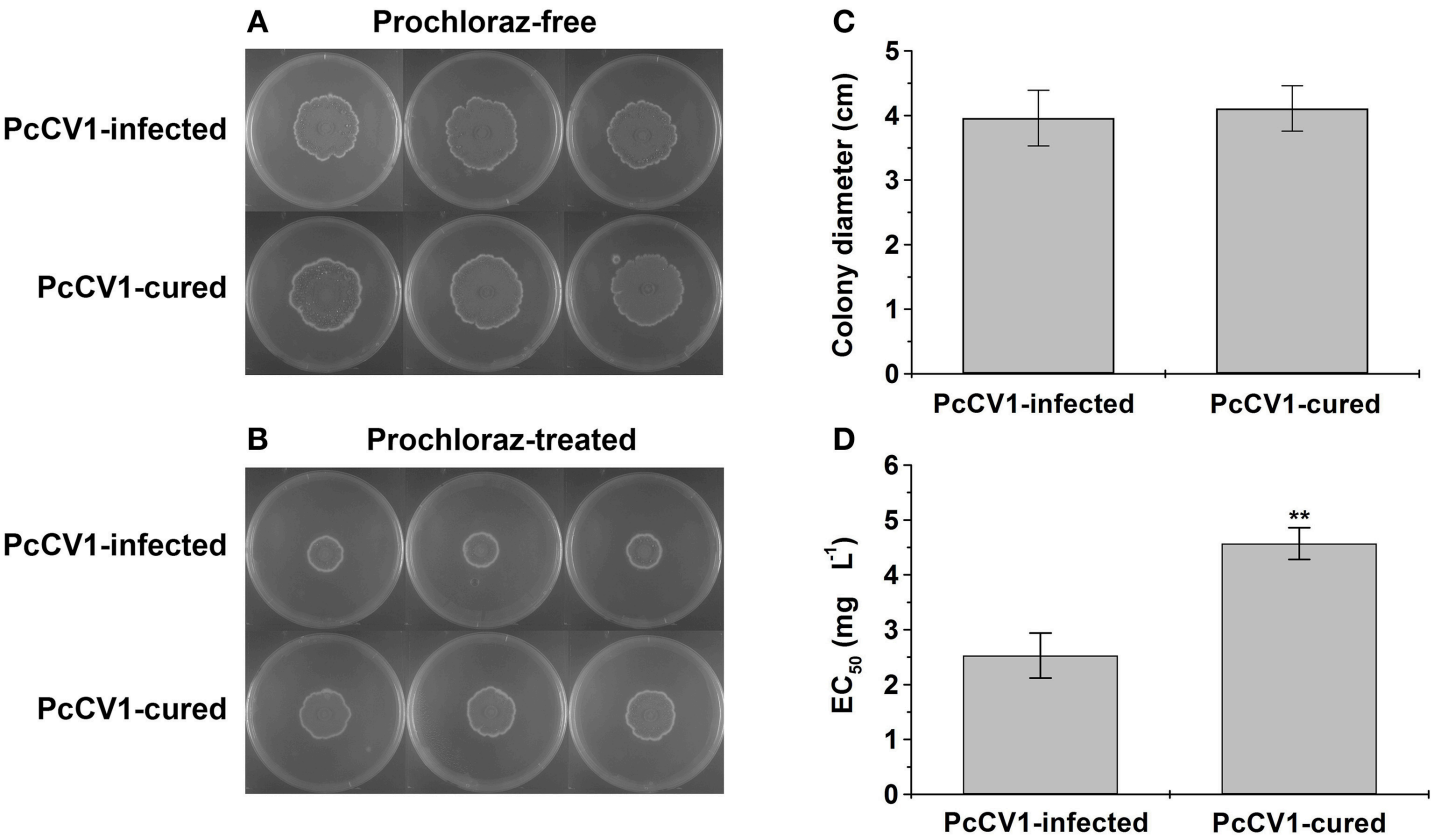
Biological effects of PcCV1-infection on its host fungus. **(A)** Effect of PcCV1-infection on vegetative growth of HS-CQ15. **(B)** Effect of PcCV1-infection on prochloraz (DMI-fungicide) resistance of HS-CQ15. The fungal strains were grown on PDA at 25°C for 7 d and photographed, with or without 5.0 mg L^−1^ prochloraz (final concentration). In panel **(A,B)**, the three plates listed in horizontal line indicate three biological replicates. **(C)** Comparison of colony diameters for HS-CQ15 (PiCV1-infected) and its PiCV1-cured progenies under fungicide-free conditions. **(D)** Comparison of EC_50_ values against prochloraz for HS-CQ15 (PiCV1-infected) and its PiCV1-cured progenies. In panel **(C,D)**, the values are shown as mean ± SD from five independent experiments (*n* = 5) with three biological replicates for each experiment, and SPSS software (version 10.0) was applied to perform statistics analysis (^**^P < 0.01).

To evaluate biological effects of PcCV1, we compared vegetative growth between PcCV1-infected HS-CQ15 and its virus-cured progenies, as well as fungicide-resistance based on PDA experiments. The colony diameters of PcCV1-infected HS-CQ15 strains were similar to those of PcCV1-cured progenies under fungicide-free conditions (Figure 2A), but obviously smaller under prochloraz conditions (Figure 2B). These results indicated that PcCV1 had little effect on the vegetative growth but deduced prochloraz resistance of its host fungus. Considering almost no effect of PcCV1 on host growth, as indicated by statistics analysis of colony diameters (Figure 2C), the present chrysovirus is not assumed as a hypovirus to decrease HS-CQ15 virulence, and this assumption needs support by citrus-based pathogenicity assessments. Here, we emphasized the PcCV1-induced decrease in prochloraz resistance for its host fungus. As shown in Figure 2D, prochloraz EC_50_ value of PcCV1-infected HS-CQ15 was 2.53 ± 0.41 mg L^−1^, significantly lower than those of PcCV1-cured progenies (4.57 ± 0.29 mg L^−1^). The similar effects were at first time reported in PdPmV1/PdNLV1-coinfected *P. digitatum* strains (Niu et al., 2018). Now we provided another evidence of mycovirus-induced fungicide-conditioned hypovirulence that would enhance drug efficacy to control citrus pathogen *P. crustosum*.

In conclusion, according to molecular features of genomic dsRNAs, homolog and phylogenetic analysis, and characteristics of their 5′- and 3′-UTRs, this report identified the first mycovirus (PcCV1) found in citrus pathogen *P. crustosum* as a variant of PcV in the *Chrysovirus* genus (*Chrysoviridae* family) and revealed that PcCV1-infection decreased prochloraz-resistance of its host fungus (HS-CQ15).

## Author Contributions

YY and DL conceived this study, acquired project funding, revised to complete final version of manuscript, and supervised all research activities. SW, ZY, and TZ designed experiments and conducted experimental procedures. QC and GL contributed to data curation and bioinformatics analysis. NL contributed to *P. crustosum* isolation and characterization.

### Conflict of Interest Statement

The authors declare that the research was conducted in the absence of any commercial or financial relationships that could be construed as a potential conflict of interest.
