# Supplementary material for: Molecular Characterization of a Chrysovirus Isolated From the Citrus Pathogen Penicillium crustosum and Related Fungicide Resistance Analysis
Source: Front Cell Infect Microbiol. 2019 May 15;9:156. doi: 10.3389/fcimb.2019.00156 (PMC6529537; doi:10.3389/fcimb.2019.00156)
Supplement: Supplementary file 1 [file Data_Sheet_1.doc]

**Supplementary materials**

**Molecular characterization of a chrysovirus isolated from the citrus pathogen *Penicillium crustosum* and related fungicide resistance analysis**

Shengqiang Wang^1*^, Zhu Yang^1*^, Tingfu Zhang^1*^, Na Li^2^, Qianwen Cao^1^, Guoqi Li^1^, Yongze Yuan^1**^, Deli Liu^1**^

^1^Hubei Key Laboratory of Genetic Regulation and Integrative Biology, School of Life Sciences, Central China Normal University, Wuhan 430079, P.R.China

^2^College of Life Science and Technology, Honghe University, Mengzi, 661199, P.R.China

*These authors contributed equally to this work.

**Correspondence: Yongze Yuan ([yuan_yongze@163.com](mailto:yuan_yongze@163.com)) and Deli Liu ([ldl@mail.ccnu.edu.cn](mailto:ldl@mail.ccnu.edu.cn); [deliliu2013@163.com](mailto:deliliu2013@163.com)).


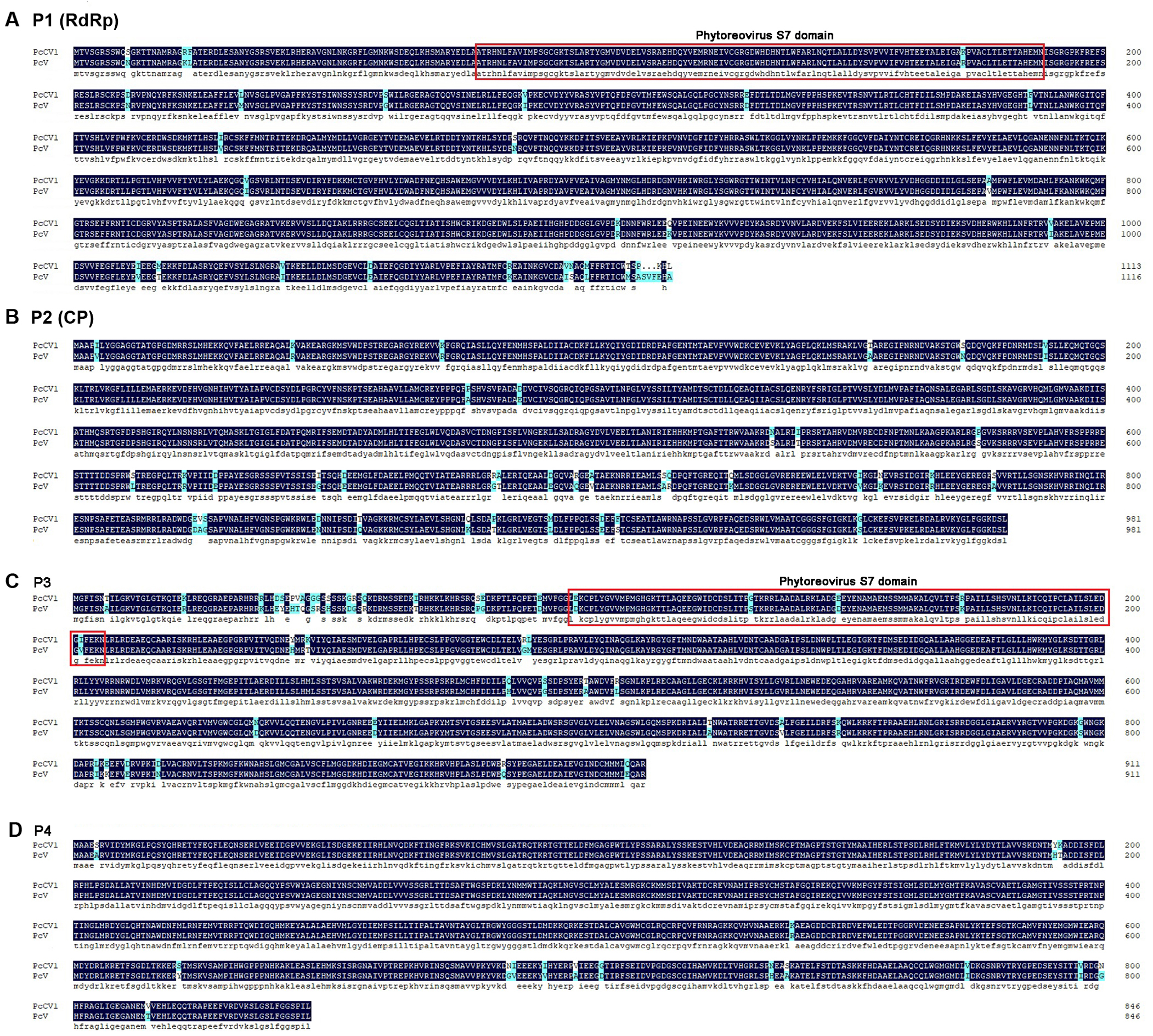


**Figure S1** Alignment analysis of putative amino-acid sequences for the corresponding genomic dsRNA-encoding proteins between PcCV1 and PcV. **(A)** P1 (RdRp) encoded by dsRNA1 of PcCV1 and PcV; **(B)** P2 (CP) encoded by dsRNA2 of PcCV1 and PcV; **(C)** P3 encoded by dsRNA3 of PcCV1 and PcV; **(D)** P4 encoded by dsRNA4 of PcCV1 and PcV. The phytoreovirus S7 domain was highlighted in red box.


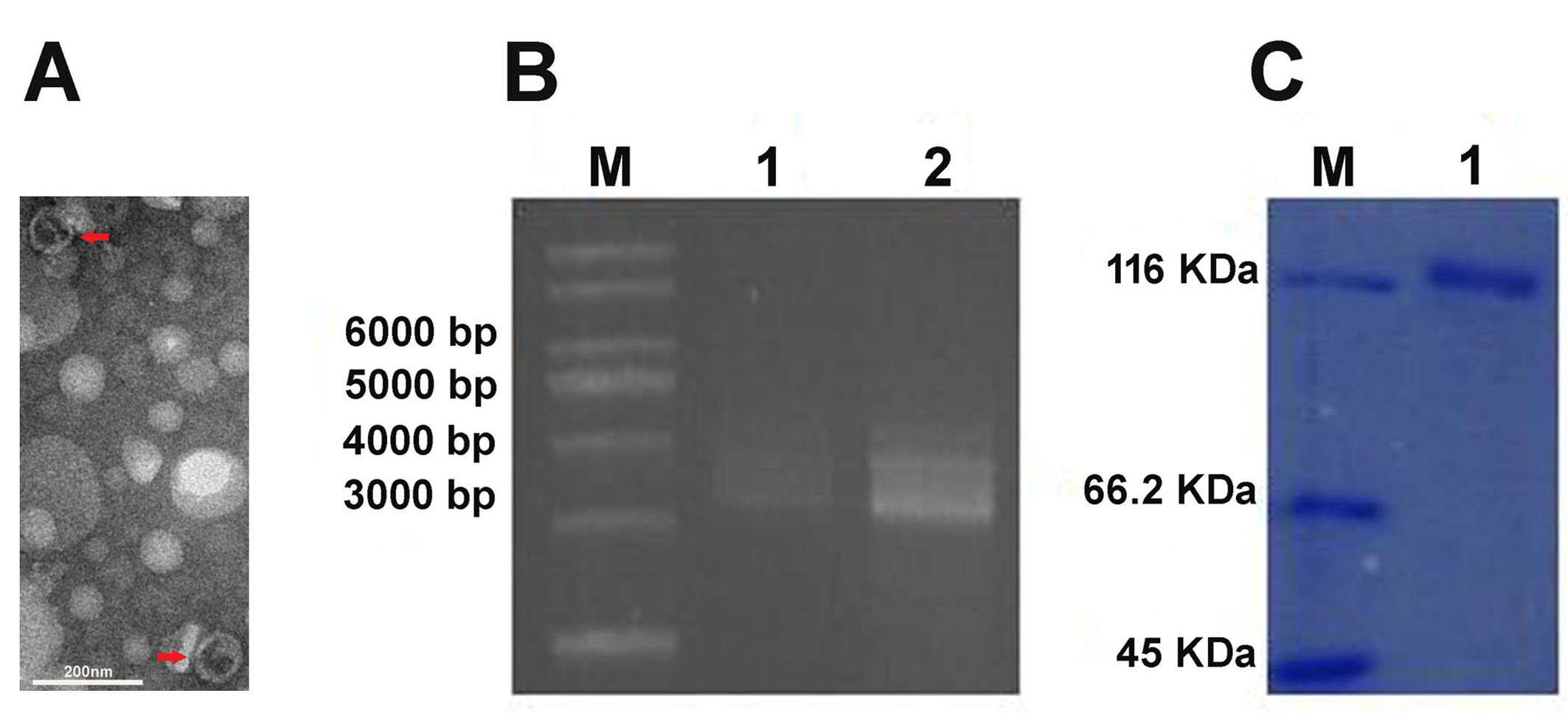


**Figure S2** Detection of viral particles (VPs) from virus-infected *P. crustosum* HS-CQ15. **(A)** Transmission electron microscopy (TEM) image of VPs purified from HS-CQ15. The VPs in the image are indicated by red arrows. **(B)** Comparison of mobility patterns of dsRNA extracted from purified VPs of PcCV1 (lane 1) and HS-CQ15 mycelia (lane 2) using 1% (w/v) agarose gel electrophoresis. Lane M indicates DNA marker DS10000 (TaKaRa, Dalian, China). **(C)** SDS-PAGE analysis of structural proteins of the purified VPs of PcCV1 (lane 1). Lane M indicates PageRuler™ Unstained Protein Ladder (Thermo Fisher Scientiﬁc, USA).


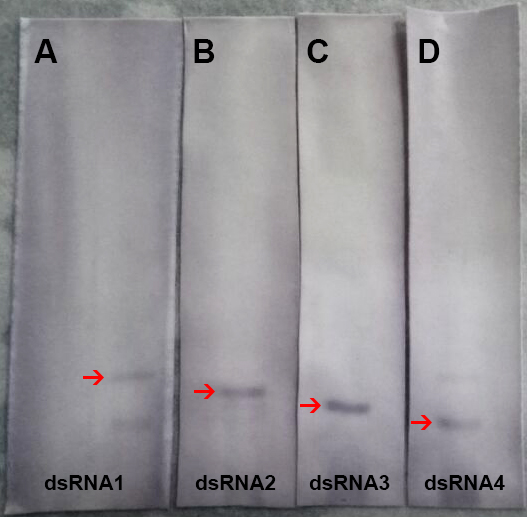


**Figure S3** The full-scan of entire original gels for the RNA blots of PcCV1 genomic dsRNAs from fungal host HS-CQ15. Panels A, B, C, and D represent the full-scan of entire original gel for dsRNA1, 2, 3, and 4, respectively, corresponding to the cropped images of blots in **Figure 1 panel B**. The dsRNAs were separated under denaturing electrophoresis conditions, blotted onto nylon membranes, and probed by DIG-labeled DNA fragments as indicated in **Figure 1 panel C**.

**Table S1** Primers used in this study.

| Name | Sequence (5’-3’) | Purpose |
| --- | --- | --- |
| P-dsRNA1-F1 | GAATAATTCCTTTGGACCTGAGG | Primers used to determine the sequence gap of the dsRNA1. |
| P-dsRNA1-R1 | AAGAATTTGCTGCACCGGATC | Primers used to determine the sequence gap of the dsRNA1. |
| P-dsRNA1-F2 | TGATCCGGTGCAGCAAATTCTT | Primers used to determine the sequence gap of the dsRNA1. |
| P-dsRNA1-R2 | CGAGCCAACACGTTGACGTAG | Primers used to determine the sequence gap of the dsRNA1. |
| P-dsRNA2-F1 | GAGAAGAAGCAGGTATTCGCCG | Primers used to determine the sequence gap of the dsRNA2. |
| P-dsRNA2-R1 | GCTCTATCCTGATATTGGCCA | Primers used to determine the sequence gap of the dsRNA2. |
| P-dsRNA2-F2 | CCAAATGGCTAGCAAATTGACCG | Primers used to determine the sequence gap of the dsRNA2. |
| P-dsRNA2-R2 | CGTTTCGCCATGCCAGAGTT | Primers used to determine the sequence gap of the dsRNA2. |
| P-dsRNA2-F3 | TAGGGGGAAGATGAGCGTAT | Primers used to determine the sequence gap of the dsRNA2. |
| P-dsRNA2-R3 | GACGGCCTTGTGACACTATAC | Primers used to determine the sequence gap of the dsRNA2. |
| P-dsRNA2-F4 | CCCTATCAGTTTCTTAGTCAATGG | Primers used to determine the sequence gap of the dsRNA2. |
| P-dsRNA2-R4 | CCTCAGCCAAGTAACTGCACAT | Primers used to determine the sequence gap of the dsRNA2. |
| P-dsRNA3-F1 | CCCATTAGGAAGCAACTCAC | Primers used to determine the sequence gap of the dsRNA2. |
| P-dsRNA3-R1 | CACTATGAATGACTGGGCT | Primers used to determine the sequence gap of the dsRNA2. |
| P-dsRNA3-F2 | GATACCTTCAAGCGTTAATGGC | Primers used to determine the sequence gap of the dsRNA3. |
| P-dsRNA3-R2 | AATTGAGGGAGCAGGGTC | Primers used to determine the sequence gap of the dsRNA3. |
| P-dsRNA3-F3 | CGGTTGTCTCTCTGCGTGT | Primers used to determine the sequence gap of the dsRNA3. |
| P-dsRNA3-R3 | CCGATGGAGCTATACCGAG | Primers used to determine the sequence gap of the dsRNA3. |
| P-dsRNA4-F1 | GAATAGCGAAAGACTGGTGGA | Primers used to determine the sequence gap of the dsRNA4. |
| P-dsRNA4-R1 | GTCGAAGATGAAACAATGGTAC | Primers used to determine the sequence gap of the dsRNA4. |
| P-dsRNA4-F2 | GCCCGGGTACTTCTCAACTAG | Primers used to determine the sequence gap of the dsRNA4. |
| P-dsRNA4-R2 | AGAGGGATCCGAGTGACTTGAC | Primers used to determine the sequence gap of the dsRNA4. |
| P-dsRNA4-F3 | CAACGCCTTCAGACCTACG | Primers used to determine the sequence gap of the dsRNA4. |
| P-dsRNA4-R3 | CTCTCTACAGTCAGTCTTGGCG | Primers used to determine the sequence gap of the dsRNA4. |
| P-dsRNA4-F4 | TTGAAATGGTTACACGACG | Primers used to determine the sequence gap of the dsRNA4. |
| P-dsRNA4-R4 | ATCCGAGTGACTTGACATCT | Primers used to determine the sequence gap of the dsRNA4. |
| Pt-dsRNA1-1 | ATGGAAGCAGATGTTTGGGACTC | Primers used to determine 5’-terminal sequence of the dsRNA1. |
| Pt-dsRNA1-2 | TGAGGGCATTATGACAGCGAAG | Primers used to determine 3’-terminal sequence of the dsRNA1. |
| Pt-dsRNA2-1 | AGACGGAAGCCTCTCGAATG | Primers used to determine 5’-terminal sequence of the dsRNA2. |
| Pt-dsRNA2-2 | CCGTACTCTTTGCAACGTCATT | Primers used to determine 3’-terminal sequence of the dsRNA2. |
| Pt-dsRNA3-1 | GTTTCAGGCTGGGGCAATGTAGG | Primers used to determine 5’-terminal sequence of the dsRNA3. |
| Pt-dsRNA3-2 | TAACTGGGCCACACGCAGAGAG | Primers used to determine 3’-terminal sequence of the dsRNA3. |
| Pt-dsRNA4-1 | GGTAGTAACCGTGTCACCCGCT | Primers used to determine 5’-terminal sequence of the dsRNA4. |
| Pt-dsRNA4-2 | CCGTTGATAGTAAACTTGTCCTGG | Primers used to determine 3’-terminal sequence of the dsRNA4. |

**Table S2** Information on the virus isolates used for sequence alignment and phylogenetic analysis of their RdRps.

| Name | Family | Genus | Abbreviation | GenBank accession no. |
| --- | --- | --- | --- | --- |
| *Amasya cherry disease associated* chrysovirus | *Chrysoviridae* | *Chrysovirus* | ACDACV | YP_001531163.1 |
| *Aspergillus fumigatus* chrysovirus | *Chrysoviridae* | *Chrysovirus* | AfuCV | CAX48749.1 |
| *Cryphonectria nitschkei* chrysovirus 1 | *Chrysoviridae* | *Chrysovirus* | CnCV1 | ACT79255.1 |
| *Fusarium oxysporum* chrysovirus 1 | *Chrysoviridae* | *Chrysovirus* | FoCV1 | ABQ53134.1 |
| *Helminthosporium* victoriae 145S virus | *Chrysoviridae* | *Chrysovirus* | Hv145SV | AAM68953.1 |
| *Penicillium chrysogenum* virus | *Chrysoviridae* | *Chrysovirus* | PcV | YP_392482 |
| *Verticillium dahliae* chrysovirus 1 | *Chrysoviridae* | *Chrysovirus* | VdCV1 | ADG21213.1 |
| *Anthurium mosaic-associated* virus-PHA | *Chrysoviridae* | Unclassified, chrysoviruses-related viruses | AmAV | [ACU11563.1](https://www.ncbi.nlm.nih.gov/protein/255367544) |
| Aspergillus mycovirus 1816 | *Chrysoviridae* | Unclassified, chrysoviruses-related viruses | AmV1816 | ABX79996.1 |
| Brassica campestris chrysovirus 1-Hubei | *Chrysoviridae* | Unclassified, chrysoviruses-related viruses | BcCV1 | AKU48197.1 |
| Botryosphaeria dothidea chrysovirus 1- LW-1 | *Chrysoviridae* | Unclassified, chrysoviruses-related viruses | BdCV1 | AGZ84312.1 |
| Colletotrichum gloeosporioides chrysovirus 1-HZ-1 | *Chrysoviridae* | Unclassified, chrysoviruses-related viruses | CgCV1 | ALW95408.1 |
| Fusarium graminearum dsRNA mycovirus-2 | *Chrysoviridae* | Unclassified, chrysoviruses-related viruses | FgV2 | ADW08802.1 |
| Fusarium graminearum mycovirus-China-9 | *Chrysoviridae* | Unclassified, chrysoviruses-related viruses | FgV-ch9 | [ADU54123.1](https://www.ncbi.nlm.nih.gov/protein/315633155) |
| Fusarium oxysporum f. sp.Dianthi virus-Fod 116 | *Chrysoviridae* | Unclassified, chrysoviruses-related viruses | FodV1 | [AKP45145.1](https://www.ncbi.nlm.nih.gov/protein/873330031) |
| Magnaporthe oryzae chrysovirus 1-A | *Chrysoviridae* | Unclassified, chrysoviruses-related viruses | MoCV1-A | [BAJ15133.1](https://www.ncbi.nlm.nih.gov/protein/304361430) |
| Magnaporthe oryzae chrysovirus 1-B | *Chrysoviridae* | Unclassified, chrysoviruses-related viruses | MoCV1-B | [BAO20927.1](https://www.ncbi.nlm.nih.gov/protein/565410873) |
| Persea americana chrysovirus-Spain | *Chrysoviridae* | Unclassified, chrysoviruses-related viruses | PaCV | AJA37498.1 |
| Raphanus sativus chrysovirus 1-D13 | *Chrysoviridae* | Unclassified, chrysoviruses-related viruses | RsCV1 | AFE83590.1 |
| *Penicillium stoloniferum* virus S | *Partitiviridae* | *Gammapartitivirus* | PsV-S | YP_052856.2 |
| *Penicillium digitatum* Gammapartitivirus 1 | *Partitiviridae* | *Gammapartitivirus* | PdGv1 |  |

**Table S3** Amino acid sequence identity between viral proteins of PcCV1 and PcV.

| Proteins | Amino acid sequence identity between PcCV1 and PcV |
| --- | --- |
| P1 (RdRp) | 97.05% |
| P2 (CP) | 96.03% |
| P3 | 95.29% |
| P4 | 98.11% |
