# Supplementary figures and images for: Molecular Characterization of a Chrysovirus Isolated From the Citrus Pathogen Penicillium crustosum and Related Fungicide Resistance Analysis
Source: Front Cell Infect Microbiol. 2019 May 15;9:156. doi: 10.3389/fcimb.2019.00156 (PMC6529537; doi:10.3389/fcimb.2019.00156)

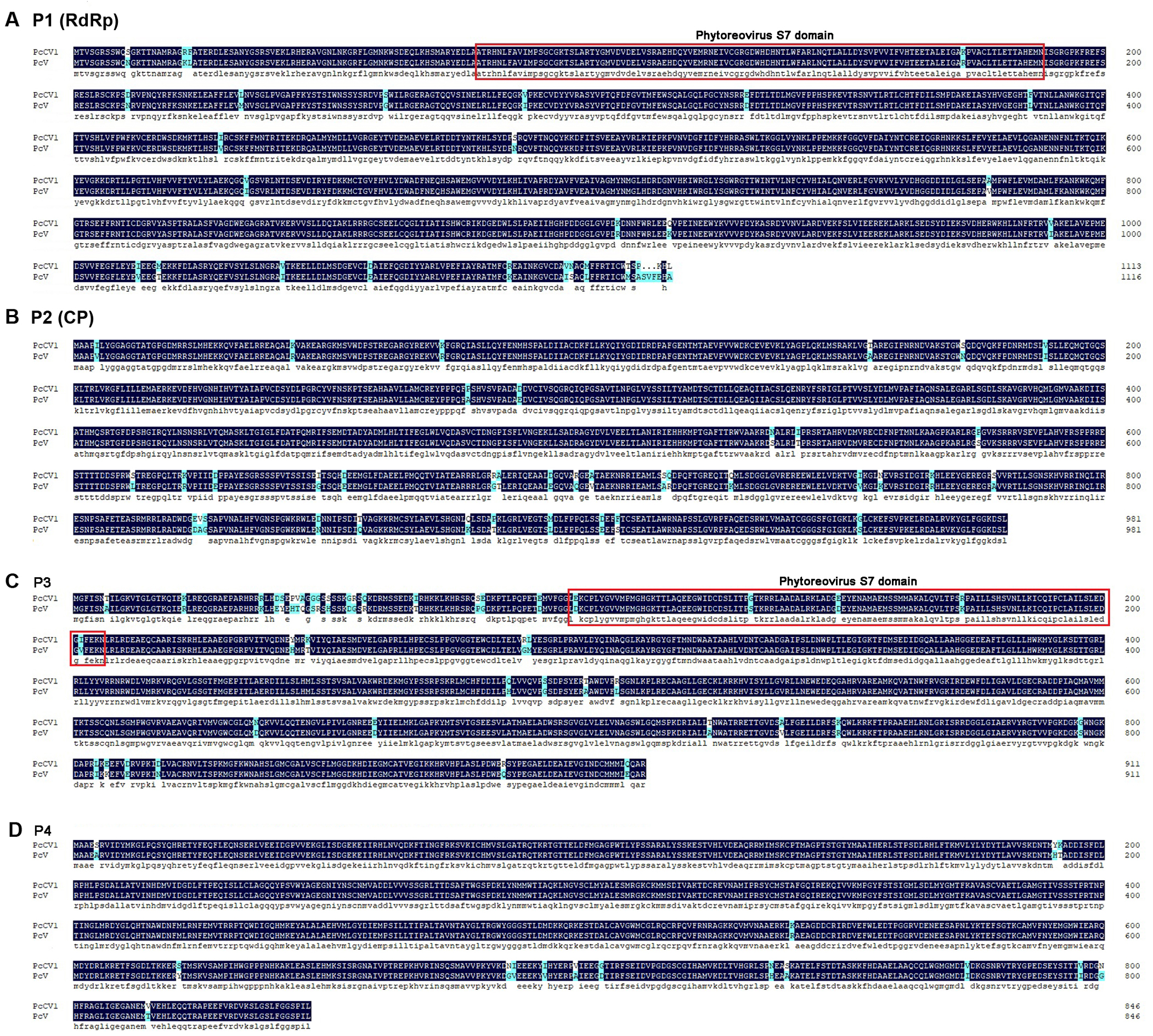

Supplement: Supplementary file 2 [file Image_1.JPEG]

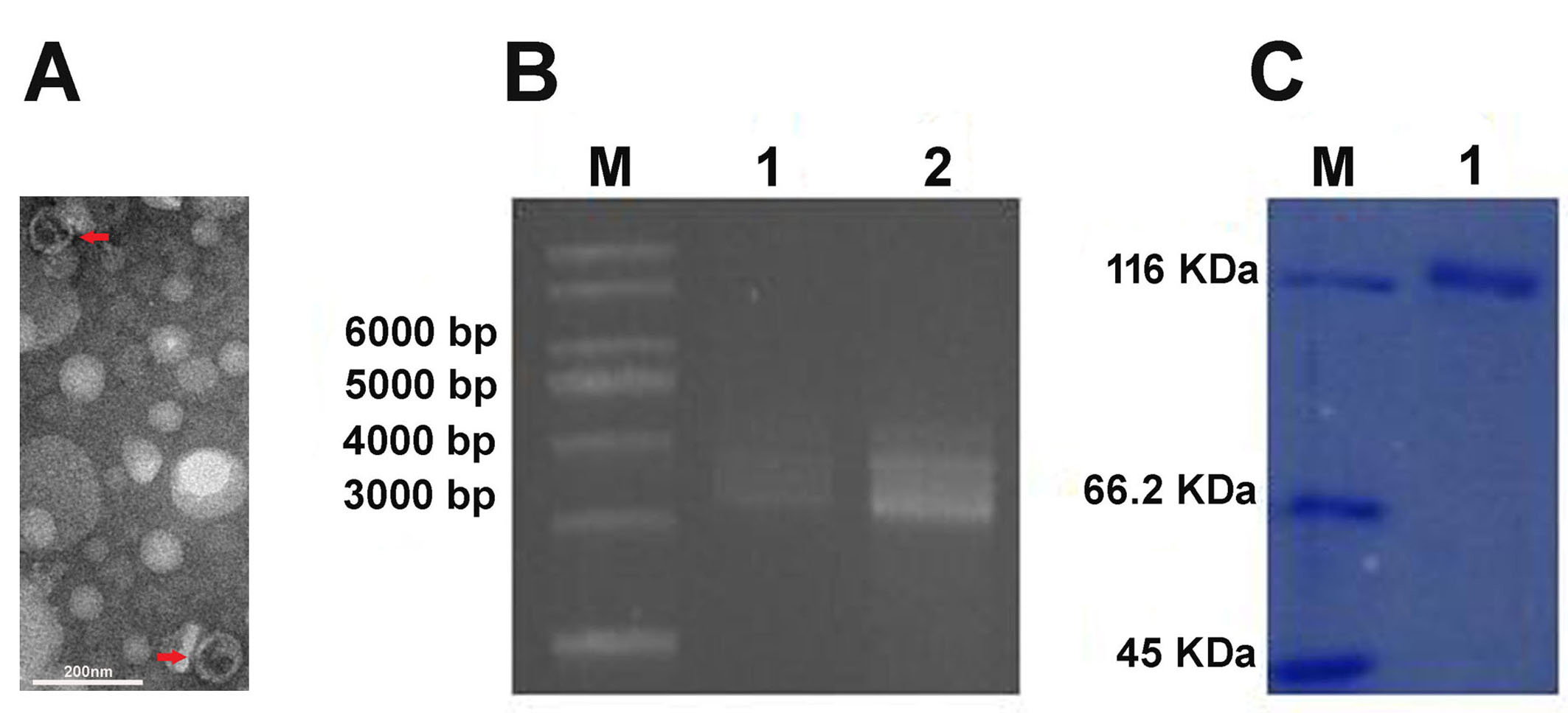

Supplement: Supplementary file 3 [file Image_2.JPEG]

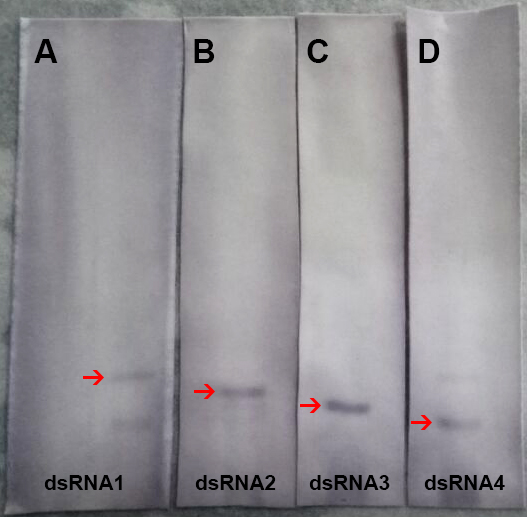

Supplement: Supplementary file 4 [file Image_3.JPEG]
